# A Case of Psoas Abscess, Mistaken during Life for Hernia

**Published:** 1890-08

**Authors:** J. L. Cunningham

**Affiliations:** Fort Worth


### A CASE OF PSOAS ABSCESS, MISTAKEN DURING LIFE FOR HERNIA.

REPORTED BY J. L. CUNNINGHAM, M. D., FORT WORTH.

Contributed to DANIEL'S TEXAS MEDICAL JOURNAL.

ON April 15, 18—, I was called to see a youth, seventeen years old, but small for his age. He had a sallow complexion and a general cachectic appearance. He was not confined to bed but walked about stiffly and with an evident effort. From the sitting position he would rise to his feet with difficulty. When laid flat on his back he could not raise himself to a sitting posture, without pulling with his hands on some object, and he could not turn over on his belly. He limped on the left leg when walking and kept the left leg more or less flexed all the time. He complained of pain in the back, and had been complaining for months. Pressure on the spinous processes of the lower dorsal and upper lumbar vertebræ elicited pain, and there was a small tumor in the left inguinal region, an inch or two above the internal abdominal ring. This tumor somewhat resembled an inguinal hernia at first sight, but closer inspection showed that it was not. There was fluctuation; there was impulse on coughing; the tumor could be made to disappear by pressure, but there was no gurgling. There was no elasticity or resistance about the tumor when pressed upon, but instead, there was a boggy, yielding feel. The tumor could be made to disappear simply by elevating the pelvis, without applying pressure to the part.

The tumor did not occupy the site of either inguinal or femoral hernia, being entirely above the inguinal and femoral canals, and having no connection with either, as I examined both and found them patulous when the hernia was down.

The cachectic appearance of the patient, taken in connection with the symptoms detailed above, particularly the great length of time he had been ailing, seemed to point unerringly to some grave constitutional trouble, and led to my diagnosing spinal caries, resulting in psoas abscess.

My prognosis was unfavorable but not necessarily hopeless. I prescribed alteratives and constructives and the *prone* position in bed. I did not evacuate the pus for fear of making matters worse by lighting up a smouldering flame. I proposed to wait and watch the effects of the treatment and be guided by developments. The family, however, were not satisfied with the diagnosis,—especially the prognosis; and without my knowledge called in Doctors A and B. Having learned this incidentally I immediately repaired to the residence of the patient, and was informed by the family that what I had heard as street talk was true, viz., that Doctors A and B had been called in; that they had condemned my diagnosis and prognosis; had instituted different treatment, and that under this new treatment the boy was rapidly improving.

I asked to be permitted to see the boy and was forthwith conducted to his room. I found him in bed, lying on his back, apparently asleep, looking more like a corpse than a living being. It was with some difficulty that he could be aroused. On applying my fingers to his wrist I counted 160 beats to the minute; pulse weak and fluttering, accompanied by a peculiar thrill that I can scarcely describe. I would here premise, that a few days previously I had read in the New Orleans Medical and Surgical Journal, a communication from Prof. Joseph Jones, in which he vividly portrayed the symptoms of heart clot, which made a profound impression on me. From the resemblance of this pulse to that described by Prof. Jones I was sure that I was in the immediate presence of a heart clot, and that the patient was in articulo mortis.

On taking up his shirt to examine the tumor, I discovered a truss adjusted to it, and was told that *the doctors had found a rupture ! That they had reduced it ! ! and that the truss I saw was to keep it up ! ! ! Mirabile dictu! Mirabile visu! Mirabilia!* I told them that this farce was now about played out,—that the boy was dying and that he would be a corpse before morning, and that I *insisted* on a post-mortem examination, in order that there might be no mistake when the fool-killer came around in the performance of his official duty.

Before breakfast the next morning I received a message that the boy was dead and the way clear for an autopsy. I immediately notified every doctor in the village (there being five, besides myself),—Doctors A and B included—inviting them to meet me at the residence of the late patient at a given hour. All put in an appearance, except Doctor A, and we immediately proceeded to business.

At the site of the tumor a sac was discovered, which, being punctured, discharged a large quantity of pus. The track of the abscess led across the rim of the pelvis in the course of the psoas muscles, which muscles were entirely absorbed, and the periosteum of the lumbar vertebræ, together with the lower dorsal, was entirely destroyed, leaving the bones bare. From the place of the tumor to the last dorsal vertebræ there appeared to be a well-worn path, reminding one of the pathway leading from one ant-bed to another. The bodies of the lower dorsal and several of the lumbar vertebra were carious but no matting together, hence, thre was no spinal curvature.

Pus in small quantity was found in the descending colon. The lobes of both lungs were hepatised, and in the left venticle of the heart was found *a heart clot*, as large as a pullet's egg, entangled in the chordæ tendinæ.

The inguinal and femoral canals were normal and patulous, *i. e.*, would admit the end of the finger pressed from without.

